# Assessment of a novel patient-specific 3D printed multi-material simulator for endoscopic sinus surgery

**DOI:** 10.3389/fbioe.2022.974021

**Published:** 2022-11-17

**Authors:** Giulia Molinari, Nicolas Emiliani, Laura Cercenelli, Barbara Bortolani, Camilla Gironi, Ignacio Javier Fernandez, Livio Presutti, Emanuela Marcelli

**Affiliations:** ^1^ Department of Otolaryngology-Head and Neck Surgery, IRCCS Azienda Ospedaliero-Universitaria of Bologna, Bologna, Italy; ^2^ Department of Experimental Diagnostic and Specialty Medicine, University of Bologna, Bologna, Italy; ^3^ eDIMES Lab-Laboratory of Bioengineering, Department of Experimental Diagnostic and Specialty Medicine, University of Bologna, Bologna, Italy

**Keywords:** endoscopic surgery, 3D printing, surgical training, patient-specific anatomy, 3D modeling, ENT surgery, augmented reality

## Abstract

**Background**: Three-dimensional (3D) printing is an emerging tool in the creation of anatomical models for surgical training. Its use in endoscopic sinus surgery (ESS) has been limited because of the difficulty in replicating the anatomical details.

**Aim**: To describe the development of a patient-specific 3D printed multi-material simulator for use in ESS, and to validate it as a training tool among a group of residents and experts in ear-nose-throat (ENT) surgery.

**Methods**: Advanced material jetting 3D printing technology was used to produce both soft tissues and bony structures of the simulator to increase anatomical realism and tactile feedback of the model. A total of 3 ENT residents and 9 ENT specialists were recruited to perform both non-destructive tasks and ESS steps on the model. The anatomical fidelity and the usefulness of the simulator in ESS training were evaluated through specific questionnaires.

**Results**: The tasks were accomplished by 100% of participants and the survey showed overall high scores both for anatomy fidelity and usefulness in training. Dacryocystorhinostomy, medial antrostomy, and turbinectomy were rated as accurately replicable on the simulator by 75% of participants. Positive scores were obtained also for ethmoidectomy and DRAF procedures, while the replication of sphenoidotomy received neutral ratings by half of the participants.

**Conclusion**: This study demonstrates that a 3D printed multi-material model of the sino-nasal anatomy can be generated with a high level of anatomical accuracy and haptic response. This technology has the potential to be useful in surgical training as an alternative or complementary tool to cadaveric dissection.

## 1 Introduction

Endoscopic sinus surgery (ESS) represents the current gold standard technique for several pathologies, ranging from chronic rhinosinusitis to benign and malignant neoplasms (Weber and Hosemann, 2015). The paranasal sinuses are an anatomically complex district, located in close relationship with noble structures, such as the brain, the orbit, and the internal carotid artery. The presence of several anatomical variants makes the surgical approaches strictly dependent on the patient’s specific configuration (Beale et al., 2009; Vaid and Vaid, 2015). Furthermore, being a one-handed technique, ESS requires hand-eye coordination and proper instruments handling in a restricted surgical area; despite technical advancements, such as the use of neuronavigation, complications from ESS can still be very serious, even fatal (Stammberger et al., 1995; Keerl et al., 1999; Humphreys and Hwang, 2015).

These peculiarities account for the importance of specific training for this type of surgery, aimed to both comprehensively understand sino-nasal anatomy and develop the required surgical skills in a risk-free environment, before operating on a patient (Acar et al., 2010; Laeeq et al., 2013; Stew et al., 2018).

The restrictions of traditional training based on cadaveric dissections have led to the investigation of alternative training methods, including the use of synthetic anatomical models created with 3-dimensional (3D) printing. The emerging advanced additive manufacturing technologies have lately expanded the possibilities of representing 3D patient-specific anatomies, which are extremely useful for both surgical planning, training (Ganguli et al., 2018; Battaglia et al., 2019; Bianchi et al., 2019; Schiavina et al., 2019; Tejo-Otero et al., 2019; Bianchi et al., 2020) and medical education (Cercenelli et al., 2022). Also, in the field of ENT surgery, 3D modeling and printing technologies allow the synthesis of patient-specific anatomical models that can be used in simulation to train on surgical steps of ESS, similarly to standard training on the cadaver (Tack et al., 2016; Sander et al., 2017). The use of 3D printing in sinus surgery is not recent, but it has been limited by some technical difficulties in printing the intricate details of the sinus anatomy and in reproducing the haptic feedback of soft tissues. New opportunities may be offered by the material jetting 3D printing technology that enables the printing of several polymers with varying properties, such as color and hardness, to be mixed at customizable ratios in a 3D space within the same model.

The aim of this study was to develop a patient-specific simulator for ESS using the 3D printing multi-material jetting technology, which reproduces both soft tissues and bony structures, thus increasing the anatomical realism and tactile feedback of the model. Moreover, a preliminary validation of this simulator as a training tool for ESS among a group of ear-nose-throat (ENT) residents and experts, is described.

## 2 Materials and methods

### 2.1 Model creation

The design and fabrication of the simulator consisted of three steps: A) Image segmentation and 3D modeling of the anatomical components; B) Design of the simulator box and the external face mold; C) 3D printing of the simulator components.

The simulator was then assembled and tested by the surgeons in the operation room.

#### 2.1.1 Image segmentation and 3D modeling

The preoperative computed tomography (CT) scan of a patient with no evidence of sino-nasal pathologies was acquired. The Digital Imaging and Communications in Medicine (DICOM) data were imported into D2P^TM^ (3D Systems Inc., Rock Hill, SC, United States), which is a certified software for medical 3D modeling. The anatomical structures of interest, i.e., the midface bony structures, the turbinates, and nasal septum were segmented in collaboration with a radiologist with expertise in head and neck anatomy. The segmented mask for the turbinates was slightly reduced, in accordance with the surgical team, to simulate the effect of decongestants used before the ESS. 3D meshes were then generated from all the segmented masks and saved in STL format; For converting the segmentation mask to 3D mesh, we used the standard settings provided by D2P software. In detail, these settings include selection of: “*Maintain original morphology*” method (to preserve all segmented voxels of the mask in the mesh file) and selection of “*Simplify and smooth*” (to decimate and apply extra smoothing to the mesh surface).

**FIGURE 1:**
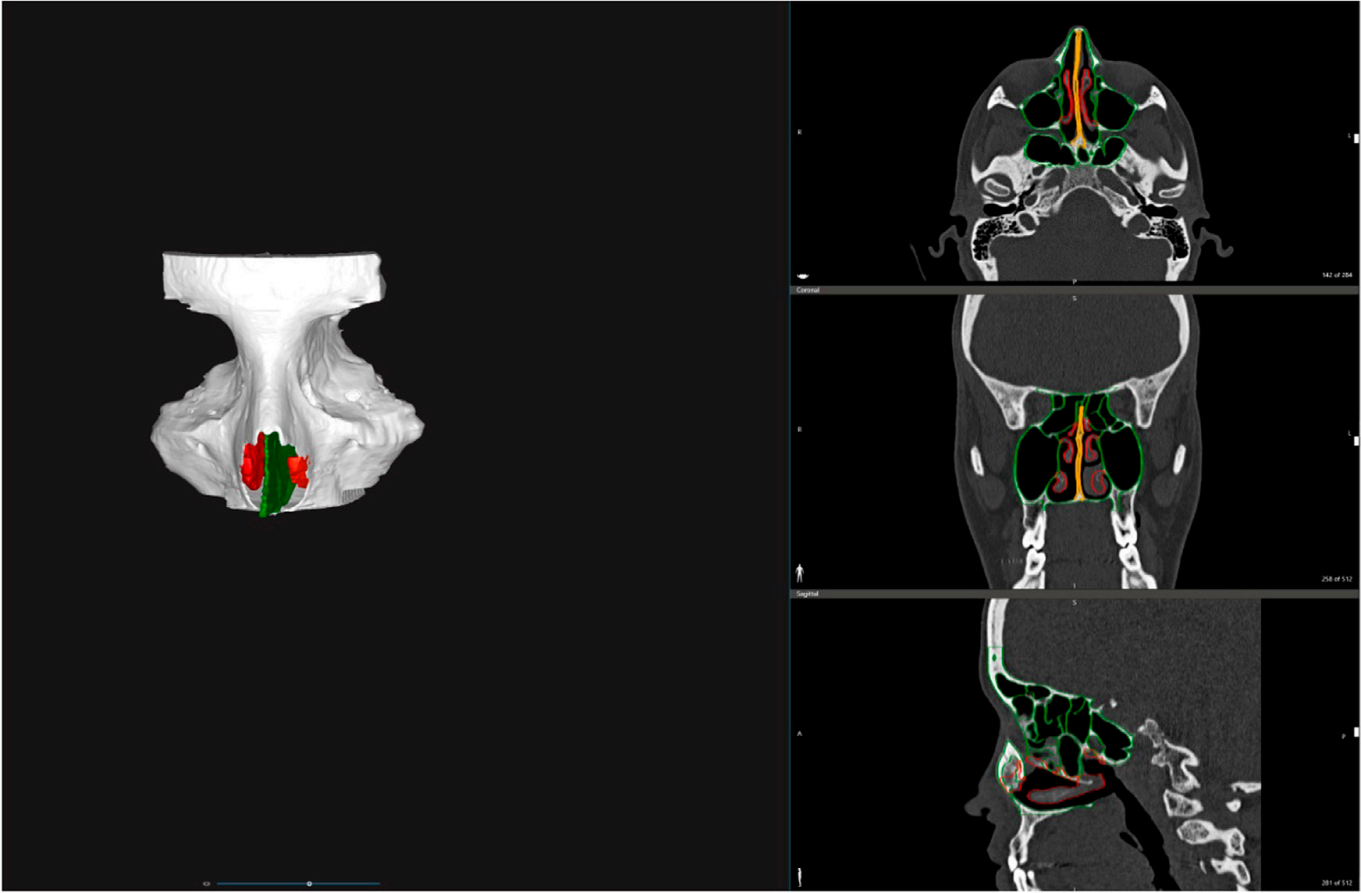
Segmentation and 3D rendering of the bony structures (white), the turbinates (red) and both bony and cartilaginous nasal septum (green).

**FIGURE 2:**
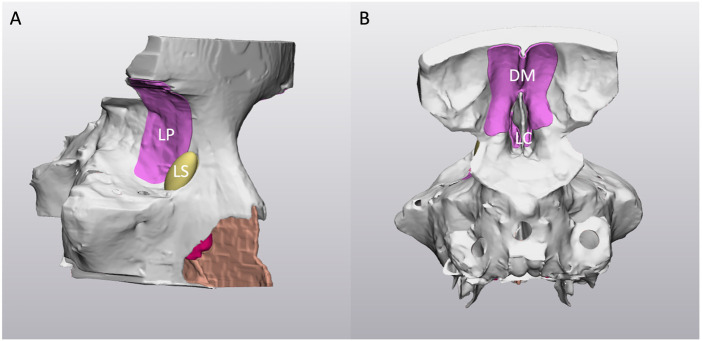
Right-lateral **(A)** and posterior **(B)** view of the virtual 3D model. Note the holes on the sphenoid sinus to allow the cleaning after the 3D printing process. **LP**: lamina papyracea; **LS**: nasolacrimal sac; **DM**: dura mater; **LC**: lamina cribra.

The meshes were imported and processed into 3-Matic Medical software (Materialise, Leuven, Belgium): all the structures were cut coronally into two sections to ease the cleaning of the internal parts after the printing process. According to the surgeons’ requests, the medial periorbital surface and the dura mater of the anterior skull base were selected as relevant anatomical landmarks for surgery, and they were enlarged in order to be more visible in the simulator. Moreover, the nasolacrimal duct and the lacrimal sac were reproduced, filling the segmented bony duct and the lacrimal sac fossa (Figures 1, 2). Finally, the inferior turbinates were hollowed with a shell of 1 mm to allow the subsequent filling with silicone, thus achieving more compressible structures. All these refinements of the 3D model were performed following the surgeon’s indications.

As final step, the Fix Wizard tool of 3-Matic software was applied to the meshes to make sure they were printable.

#### 2.1.2 Design of the simulator box and the external face mold

A box including the reconstructed patient-specific paranasal sinuses model was created using the open-source software FreeCAD. The box was designed as a hollow rectangle combined with a larger base in order to secure it to the operating table during the simulation.

The portion of the facial skin was segmented in D2P^TM^, then converted into a mesh and exported as STL file to be further processed in MeshMixer software (Autodesk Inc., CA, US).

**FIGURE 3:**
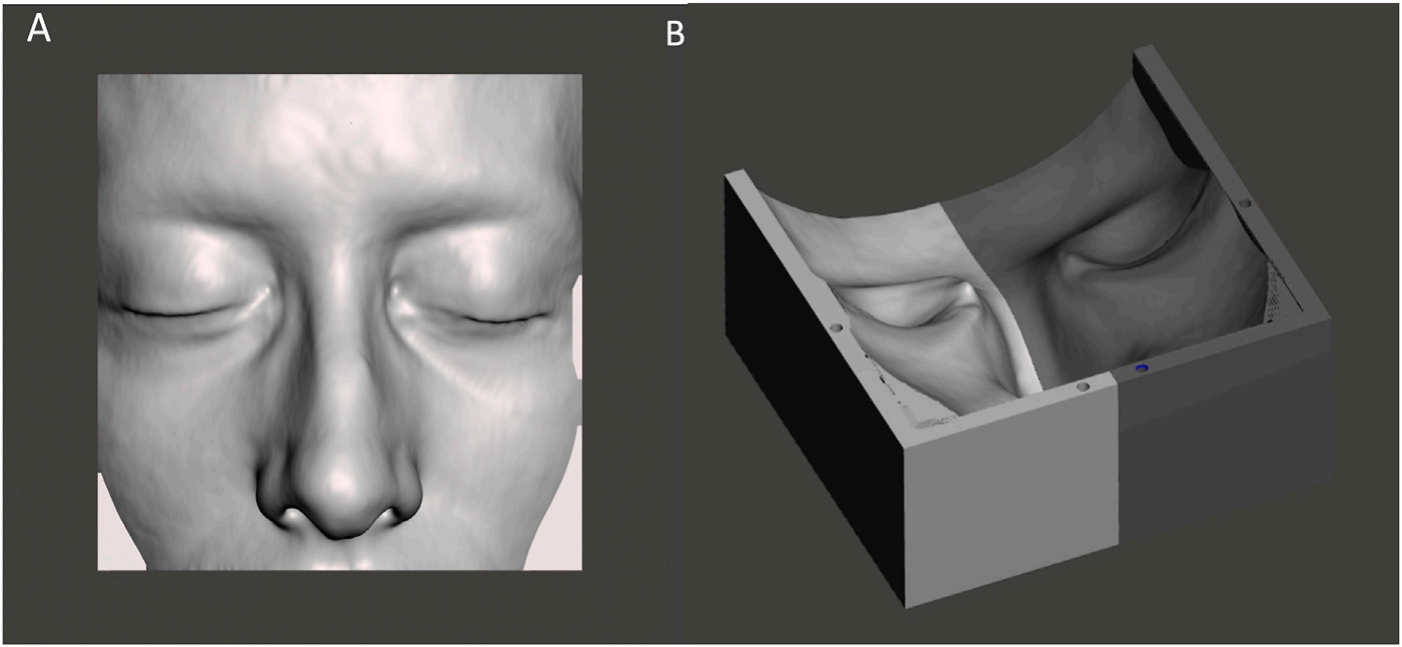
The STL file of the patient-specific portion of skin **(A)** used to create the virtual model of the facial mold **(B)**.

Using the negative of the facial skin model, a rectangular mold was designed to allow the subsequent silicone casting (Figure 3).

Lastly, the box was provided with a closing lid that was designed in FreeCAD. The patient-specific portion of skin was combined with the lid to let its borders assume the shape of the patient-specific face.

#### 2.1.3 3D printing of the simulator components

The patient-specific model of the paranasal sinuses was printed using the J720 Dental 3D printer (Stratasys Ltd., Eden Prairie, MN). This printer is based on material jetting technology, a process that resembles inkjet paper printing since the material is dropped through small diameter nozzles. In this case, the printing material is a photopolymeric resin subsequently hardened by a UV lamp.

**FIGURE 4:**
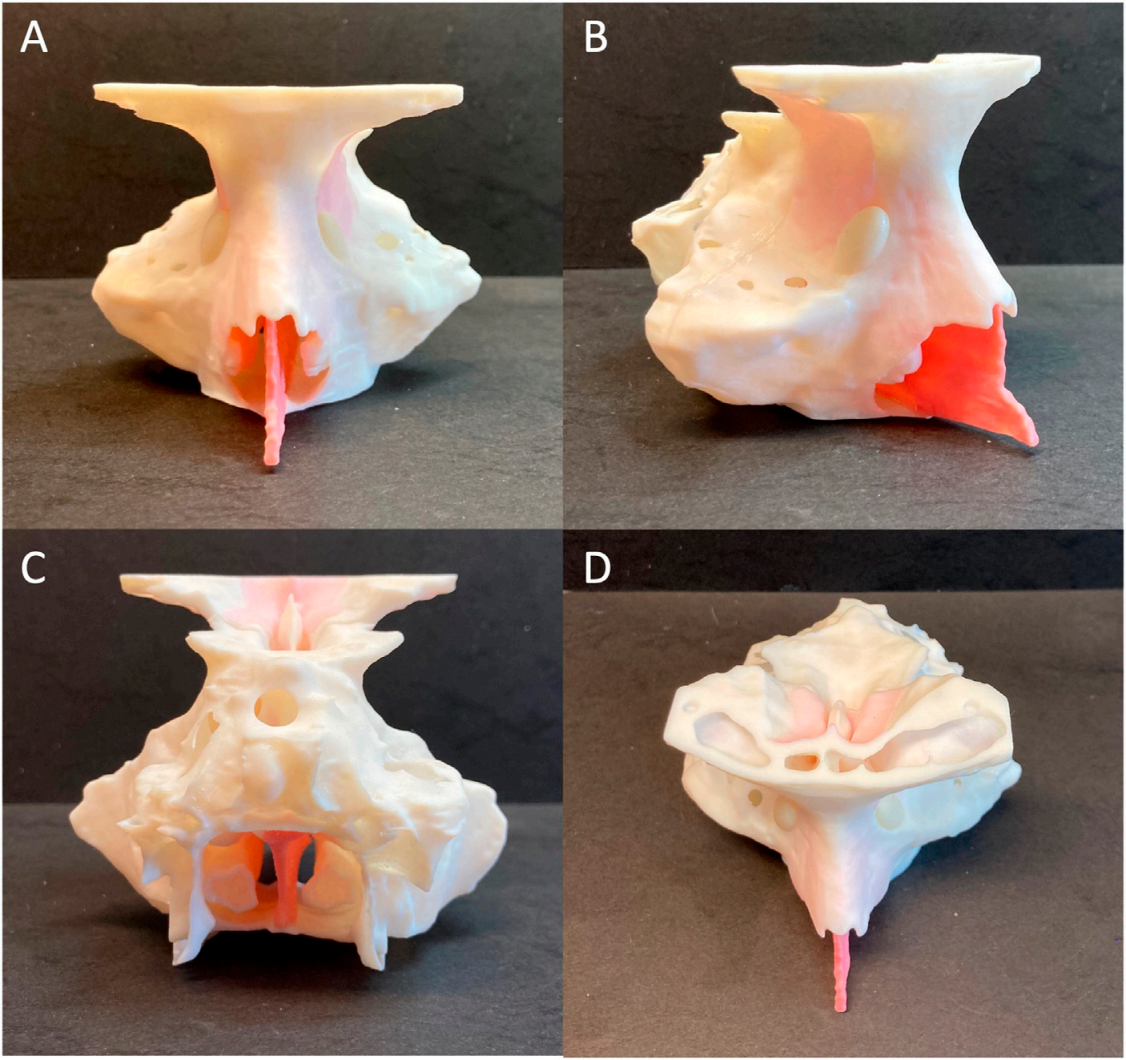
Frontal **(A)**, right-lateral **(B)**, posterior **(C)** and superior **(D)** views of the 3D printed patient-specific model of paranasal sinuses.

In detail, the bony structures of the simulator were 3D printed using a rigid white opaque material (VeroWhitePlus resin), which was previously used for reproducing bones in other 3D printed phantoms of thorax, pelvis and prosthetic fingers (Cabibihan, 2011; Jahya et al., 2013; Mayer et al., 2015). Considering the cartilage mechanical properties, that lie between bone and soft tissue (Richmon et al., 2005; Richmon et al., 2006; Griffin et al., 2016), the cartilaginous nasal septum was printed *via* a mixing of rigid resins (VeroMagenta) and a rubber-like material from the Polyjet photopolymer family (Agilus30) with a resulting Shore-A 50. Also, the membranes replicating the orbital content and the dura mater as well as the middle, the superior turbinates, and the inferior turbinates were printed combining Agilus30 and rigid resin VeroMagenta with Shore-A 50 and Shore-A 30 respectively. The lacrimal sac was printed by mixing VeroYellow and Agilus30 with a Shore-A 35 (Figure 4). Particularly, the specific hardness for turbinates and the lacrimal sac was chosen in agreement with the surgeons, after several trials on prototypes.

Regarding the other components of the simulator, the box and the lid were both printed using a stereolithography (SLA) 3D printer (Form 3, Formlabs, Somerville, MA, United States) with White resin.

The components of the patient-specific facial skin mold were also printed using Form 3 SLA printer but with a Clear resin so that, during the silicone injection, both the level of silicone and the presence of air bubbles could be seen.

#### 2.1.4 Silicone casting and assembly of the simulator

**FIGURE 5:**
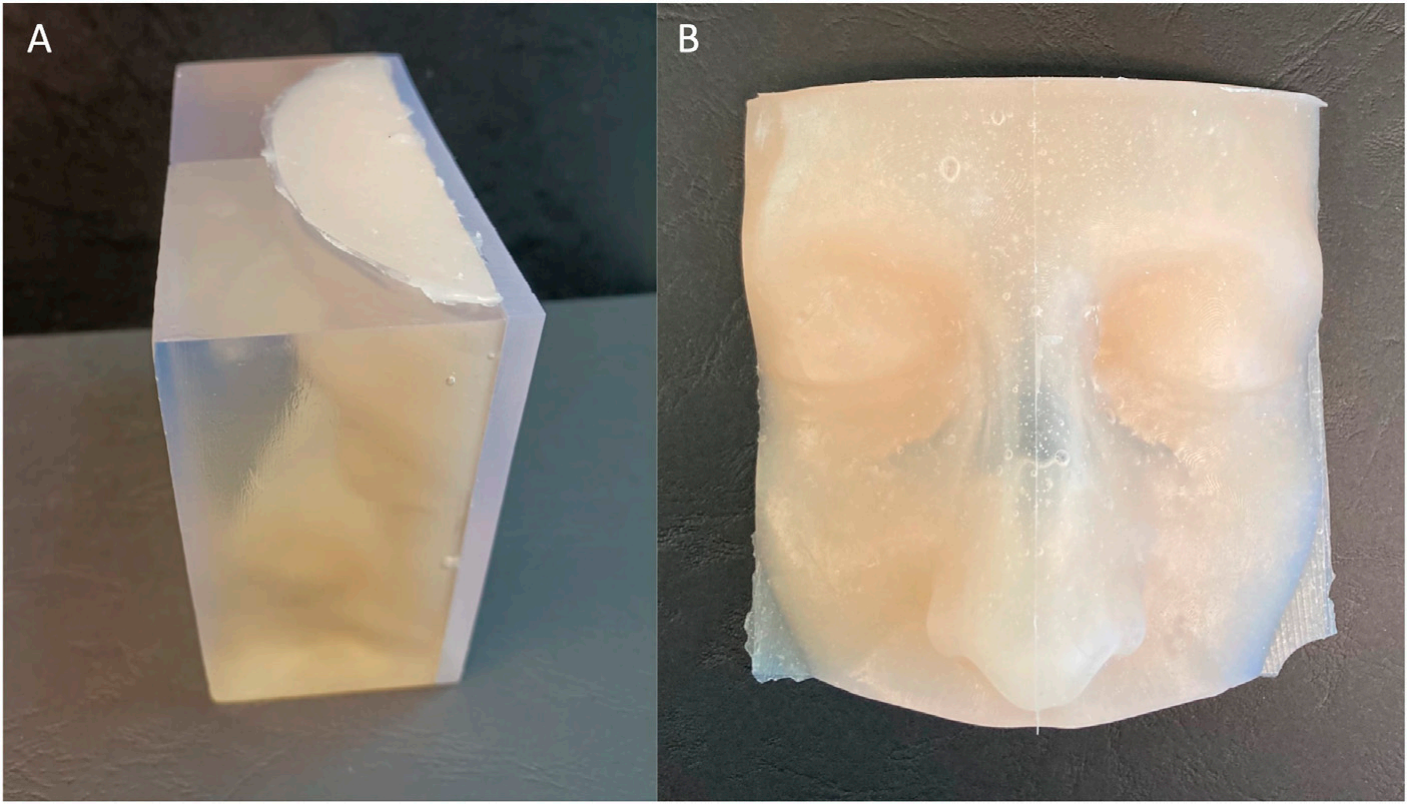
External view of the facial skin mold filled with silicone **(A)** and the obtained physical model after silicon casting **(B)**.

The patient-specific facial skin was realized by a silicone casting through the mold: the selected silicone was Dragon Skin FX-Pro (Smooth-On Inc., Texas, United States) which is a bi-component silicone frequently used in literature to reproduce human skin and surgical simulation (Francesconi et al., 2015; Teyssier et al., 2019; Ock et al., 2020; Ock et al., 2021). The silicone is available as a liquid that is separated in two components, that were mixed in ratio 1:1 by volume; then some pigments (Silc-Pig, Smooth-On Inc., Texas, United States) were added to achieve the realism of the final product (Figure 5).

**FIGURE 6:**
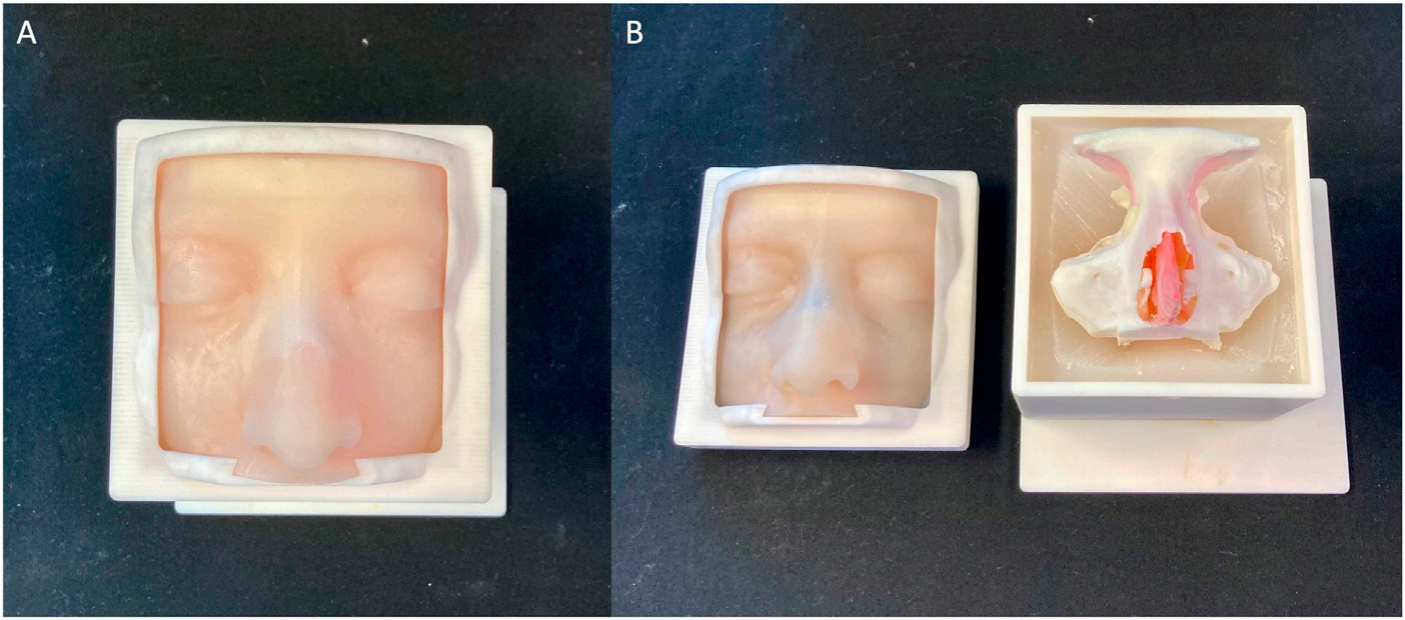
The final assembly of the simulator **(A)** and the view of the internal part **(B)**.

After the cleaning of the two parts of the model, the inferior turbinates were filled up with Dragon Skin FX-Pro, to make them more compressible. The two parts were then glued together shaping the entire model. To replicate the nasal mucosa, some layers of silicone were added over the nasal septum. Lastly, the skin portion was placed on the lid and the box was closed (Figure 6).

#### 2.1.5 Augmented Reality feature

As an additional feature, we implemented an Augmented Reality (AR) view to be integrated into the simulator. This allows a “hybrid” training, i.e. the fusion of digital and physical tools, that may improve the potentialities of the simulator.

**FIGURE 7:**
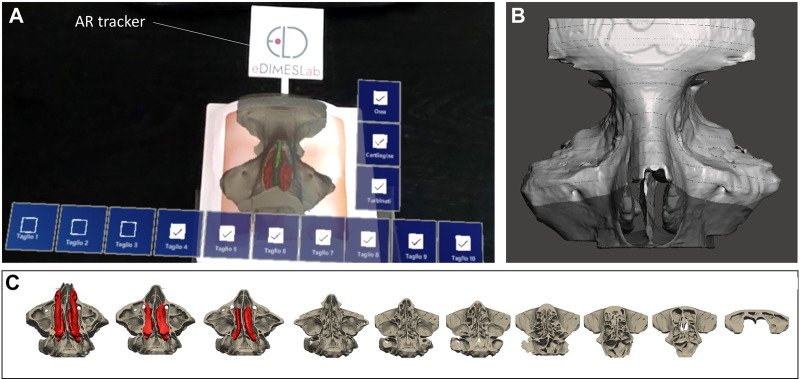
The AR view as displayed to the surgeon *via* HoloLens 2 smart glasses **(A)** to generate the holographic overlays of anatomical inner structures and cutting planes **(B,C)** on the 3D printed simulator.

The anatomical virtual models and some cutting planes planned by the surgeon on the reconstructed anatomy (Figures 7B,C) were imported into Unity 3D (Unity Technologies, San Francisco, CA, United States), a software with a specific development kit for creating AR apps (Vuforia Engine package, PTC, Inc., Boston, MA, United States).

To track the physical simulator, we designed and printed a tracker with a well-contrasted texture, which was then anchored to the external box of the simulator (Figure 7A).

The tracking algorithm and the registration between the digital models and the real scene were implemented using the Image Target function of Vuforia engine: the engine detects the image texture of the tracker by comparing extracted natural features from the camera image against the known image target (i.e., the texture previously selected for the tracker). Once the image target is detected, Vuforia engine will track the image target on the tracker and augment the scenario with digital content anchored to the target itself.

The AR application was built as a UWP (Universal Windows Platform) app deployed on Microsoft HoloLens 2 smart glasses. The user just needs to look at the tracker on the simulator while wearing the AR glasses, until the image target matching occurs. After that, tracking of the real object and virtual content overlay can begin.

The AR application generates holographic overlays on the physical model, by rendering the underlying anatomical structures (i.e., turbinates, nasal septum), not visible from the outside of the simulator, together with the cutting planes which may help the trainee in understanding the deep anatomical structures while navigating the physical model with instruments (see Supplementary Video S1).

Interactable user interface toggles (check boxes) were added to turn off and on the rendering of each virtual anatomical structure and cutting planes. In particular, cutting planes following progressive lines perpendicular to the nasal bones were created to convey the anatomical configuration that the surgeon has to bear in mind during the endoscopic approaches to the frontal sinus (commonly considered by surgeons in training a highly complex region to reach endoscopically). The possibility to virtually remove the middle turbinate allows for realistic visualization of the intraoperative field across the steps to performing DRAF procedures.

Voice commands to show/hide the virtual structures were also implemented in order to provide a completely hands-free AR application. (see Supplementary Video S1).

### 2.2 Validation of the simulator as a training tool for ESS

The process to assess the fidelity and utility of the simulator as a training tool for ESS consisted of two phases which are described below.

#### 2.2.1 Phase one: Face and anatomical content validation

First, the anatomical realism of the nasal cavity and the reproducibility of the instruments handling inside it were evaluated.

**FIGURE 8:**
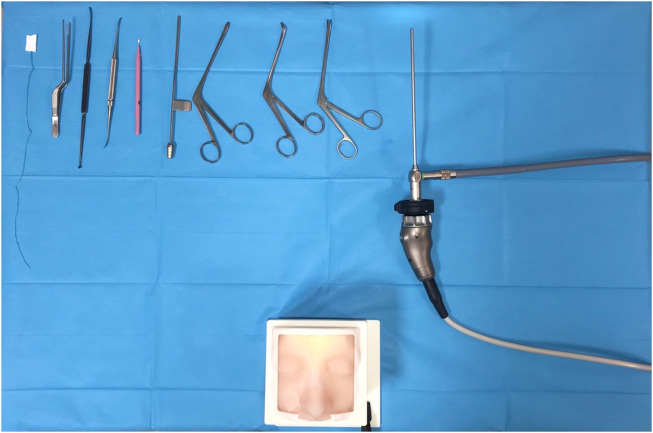
Simulation setting and instruments for non-destructive tasks.

**FIGURE 9:**
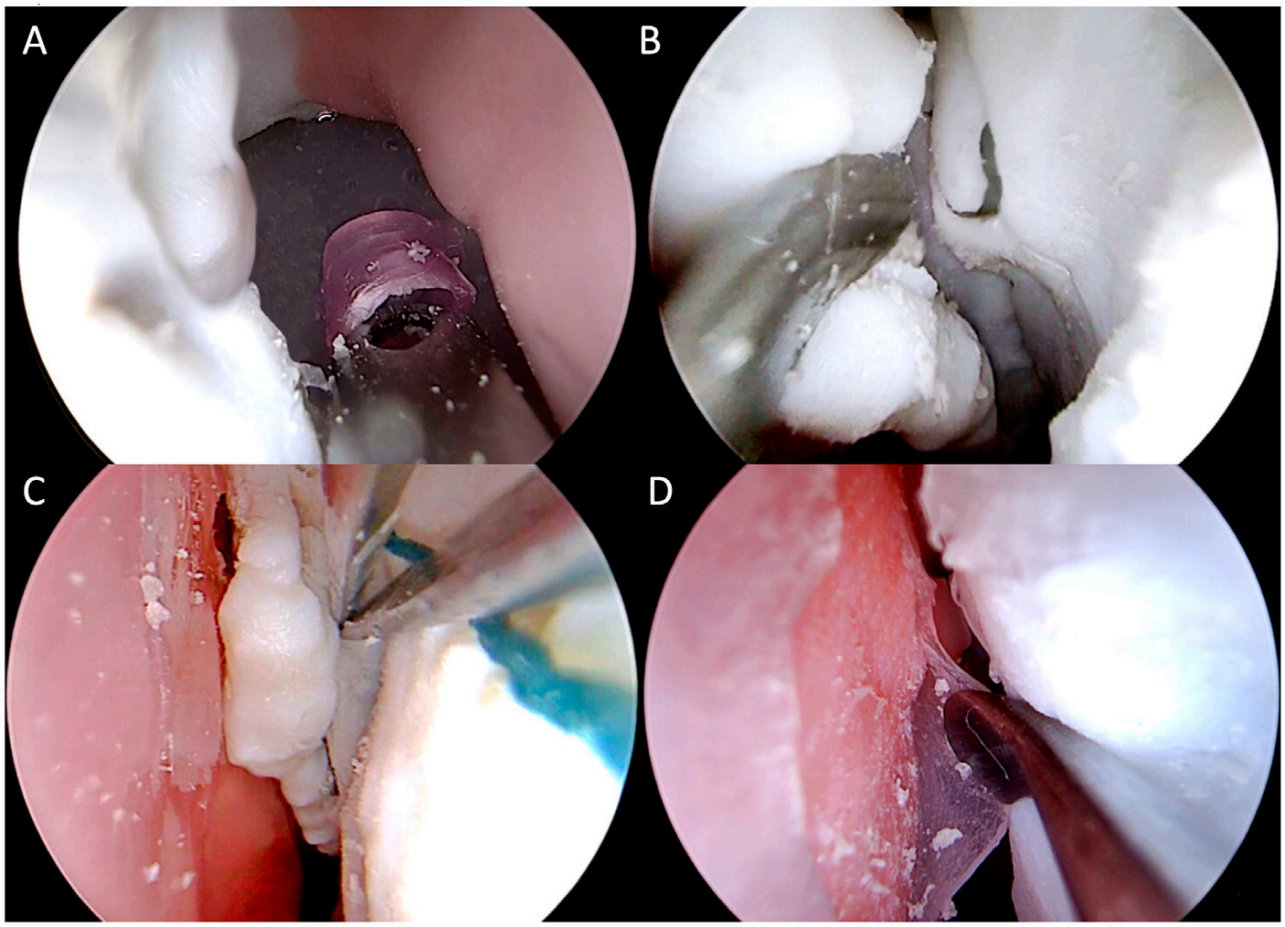
Intraoperative endoscopic images of non-destructive tasks. **(A)** removal of a plastic foreign body from the right choana; **(B)** medialization of the left middle turbinate; **(C)** insertion of a cottonoid into the left middle meatus; **(D)** detachment of the simulated septal mucosa from the underlying nasal septum of the left nasal cavity.

This phase involved 12 participants with different expertise levels: 3 ENT residents from the third and fourth year of residency, 3 young ENT specialists (less than 3 years of practice), and 6 ENT surgeons experienced in ESS (more than 10 years of practice). Each participant was asked to perform the following non-destructive tasks on the simulator using the standard instruments (Figure 8, Figure 9), in a time-trial mode:

1 Identification of normal anatomy landmarks on one nasal cavity (the inferior, middle and superior turbinate, the inferior, middle and superior meatus, the choana, the uncinate process and the hiatus semilunaris, the sphenoid ostium);
2 Removal of a foreign body (small plastic segment) from the choana;
3 Medialization of the middle turbinate and insertion of a cottonoid into the middle meatus;
4 Incision of the simulated nasal septum mucosa with a curved scalpel and its detachment from the underlying simulated cartilage with a Cottle dissector-elevator.

The completion of each task (yes/no) and the time needed to complete it were annotated by an external observer not involved in this phase (N.E.). A 5-point Likert scale questionnaire evaluating the anatomical, haptic feedback and utility of the simulator for training was filled in by each participant immediately after performing the non-destructive tasks (Supplementary Table S1).

#### 2.2.2 Phase two: Task-specific content validation

This phase was aimed at assessing the feasibility of different ESS procedures on the simulator and its potential as a training tool for ESS. Only four expert surgeons participated in this second phase. They were asked to simulate all the basic procedures of ESS, namely uncinectomy, maxillary antrostomy, anterior and posterior ethmoidectomy, sphenoidotomy (Supplementary Video S2) and DRAF I procedure, on previously untouched models (one side per surgeon). Dacriocystorhinostomy was also required, either as the first or last surgical step (Supplementary Video S3). Two of the four surgeons were asked to complete frontal sinus surgery with DRAF IIa, IIb, and eventually DRAF III (Supplementary Video S4) at the end of dissections on both sides.

After completing these tasks, the experts were required to fill out the task-specific questionnaire (Supplementary Table S2), using a Likert 5-point scale. A space for free comments and/or suggestions for improving the simulator was available at the end of the form.

### 2.3 Statistical analysis

All measured times needed to complete the tasks were reported as mean values and standard deviation (SD). *t*-test for unpaired data was used to compare mean times for each pair of participant groups (residents vs young experts, young experts vs experts, residents vs experts) for each task.

All statistical tests were performed using SPSS software, version 26.0 (SPSS Inc., Chicago, IL, United States), and a *p*-value of <0.05 was considered statistically significant.

## 3 Results

### 3.1 Face and anatomical content validation

**FIGURE 10:**
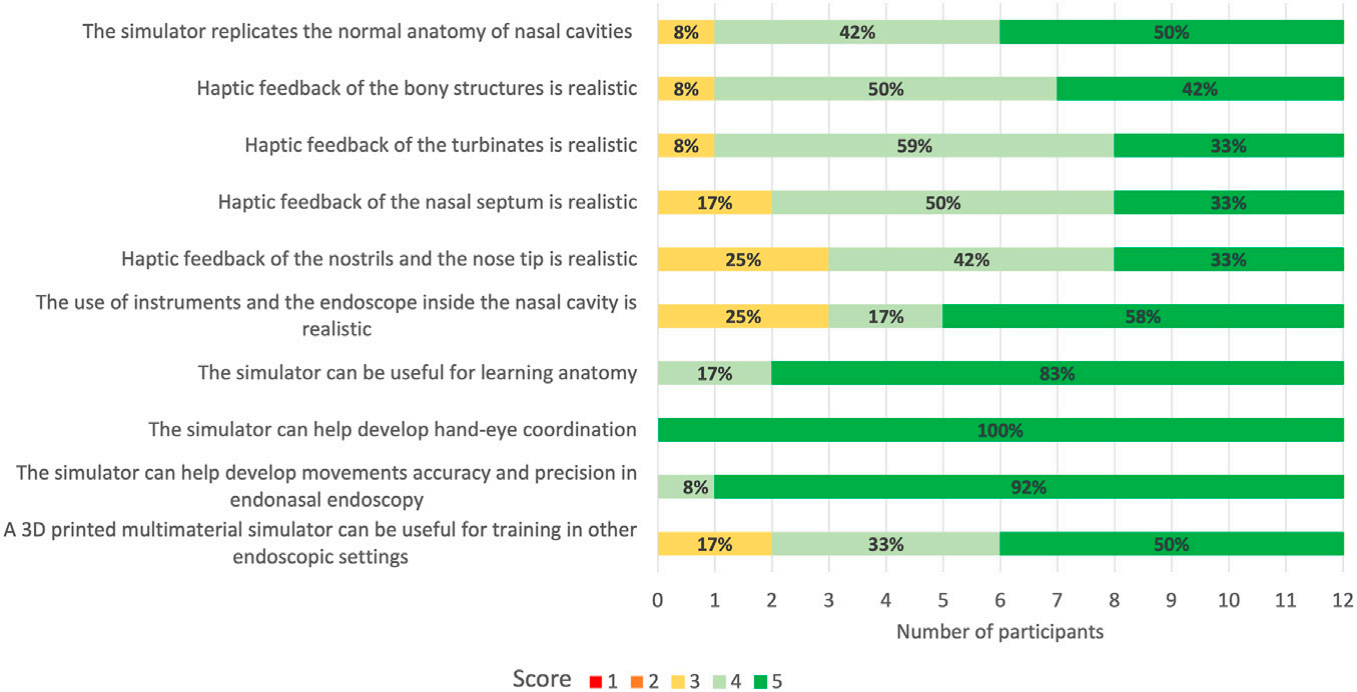
5-point Likert scale rating from face and anatomical content validation questionnaire. 1: strongly disagree; 2: disagree; 3: neutral; 4: agree; 5: strongly agree. In bold are reported the percentages for the different ratings obtained for every question.

**FIGURE 11:**
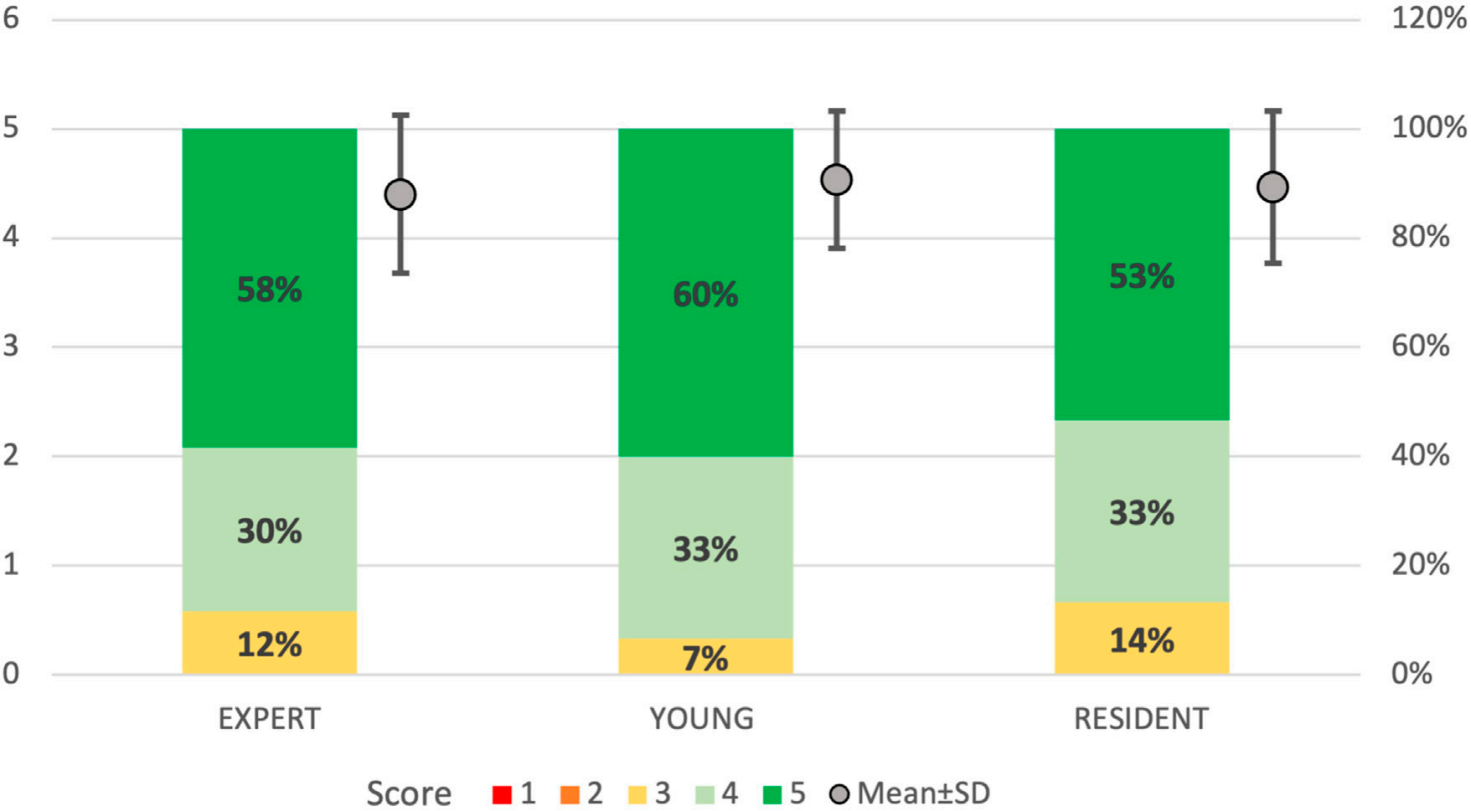
5-point Likert scale ratings divided by groups of participants. “Resident”: ENT residents from the third and fourth year; “Young”: ENT specialists with less than 3 years of practice; “Expert”: experienced ENT surgeons with more than 10 years of practice. 1: strongly disagree; 2: disagree; 3: neutral; 4: agree; 5: strongly agree. In bold are reported the percentages for each rating given by the different members of the groups. In gray dots are reported the mean and standard deviation of the scores obtained for every group.

All the 12 participants completed the face and anatomical content validation of the model and answered the survey. The non-destructive tasks were accomplished by 100% of participants (Figure 9). The survey results are reported in detail in Figure 10 and the scores stratified per group of participants are shown in Figure 11.

A total of 92% (*n* = 11) of the participants agreed or strongly agreed about the high anatomical fidelity of the simulator and the tactile feedback of both the turbinates and bony structures. On the other hand, 25% (*n* = 3) of the participants were neutral about the tactile feedback of the nostrils and nose tip and the use of instruments and endoscope inside the cavities. All the participants gave positive feedback (100% agreed or strongly agreed) regarding the role of the simulator in learning anatomy and improving accuracy and precision in endoscopic settings. Moreover, the utility of the simulator in developing hand-eye coordination received the highest scores (100% strongly agreed) from all responders.

**FIGURE 12:**
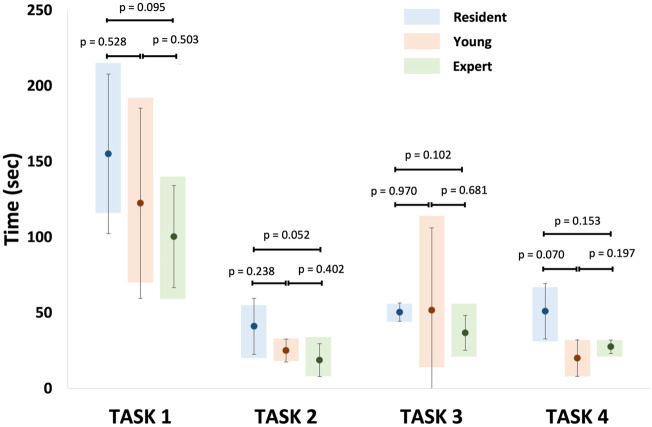
Representation of mean recorded operative times for each non-destructive tasks per group. Task 1: identification of normal anatomy landmarks; Task 2: removal of a foreign body (small plastic segment) from the choana; Task 3: medialization of the middle turbinate and insertion of a cottonoid into the middle meatus; Task 4: incision of the simulated nasal septum mucosa.

Figure 12 shows the minimum, maximum, mean, and standard deviation of the operative times recorded for each non-destructive task per groups (resident, young, and expert). Despite no statistical significance was found, results suggested that the experts completed the tasks faster than the residents.

### 3.2 Task-specific content validation

**FIGURE 13:**
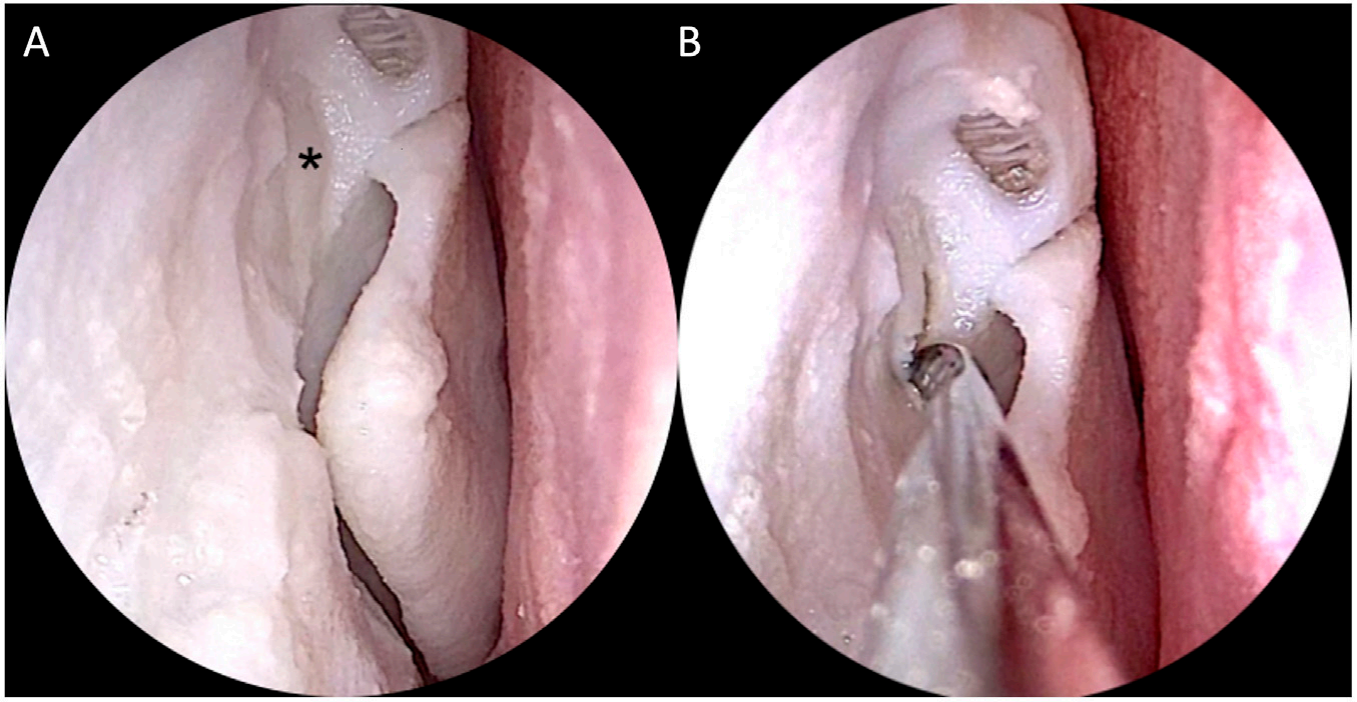
Simulation of right endoscopic dacryocystorhinostomy. **(A)** Exposure of the lacrimal sac (black asterisk); **(B)** Incision of the sac with the sickle knife.

**FIGURE 14:**
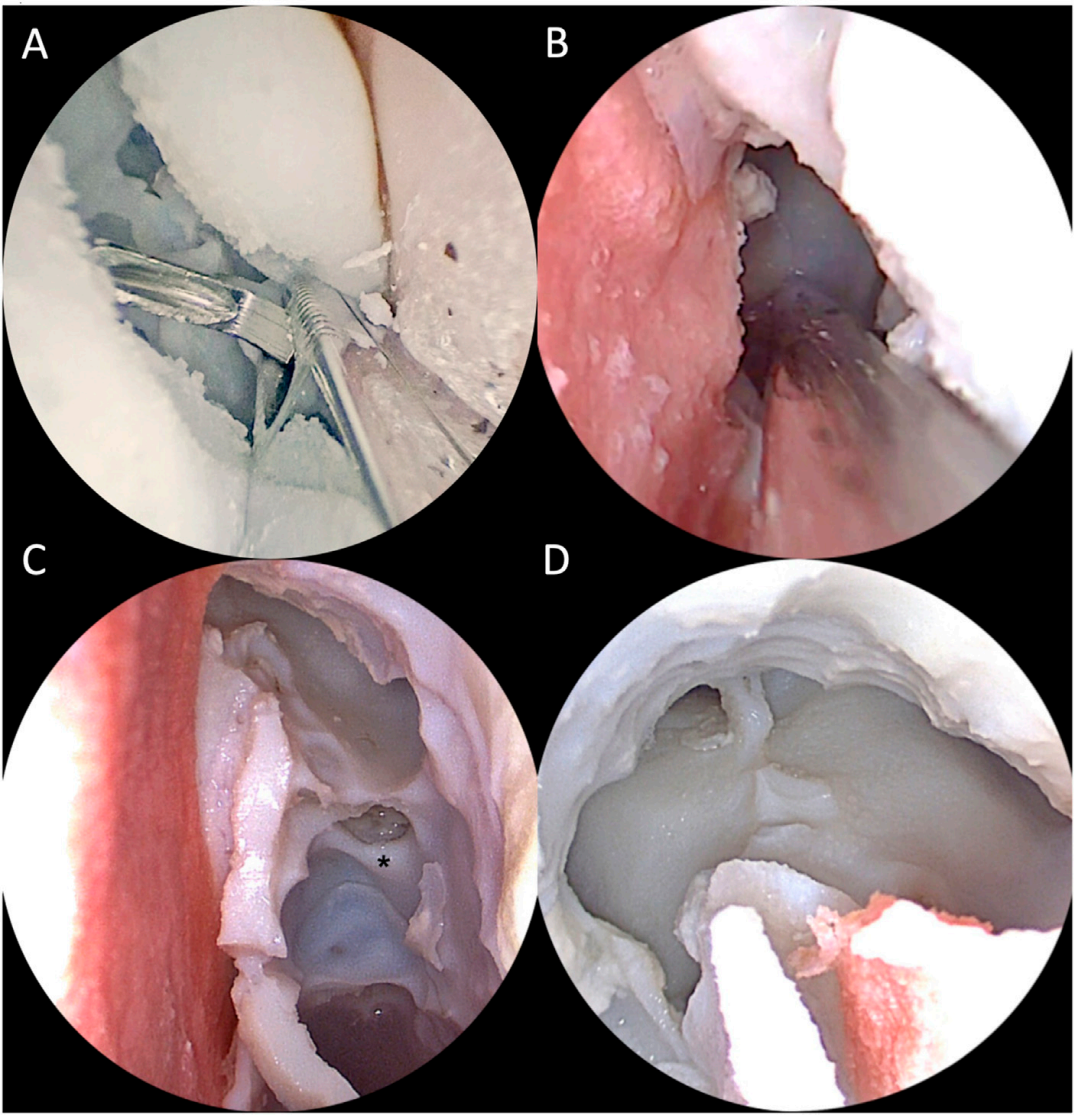
Intraoperative view of simulated endoscopic sinus surgery steps on the 3D printed model. **(A)** Right middle meatal antrostomy with backbiter forceps; **(B)** Left paraseptal sphenoidotomy; **(C)** Left approach to the frontal sinus with a 30° optic. The middle turbinate has been removed and the anterior skull base exposed. The black asterisk indicates the anterior ethmoidal artery canal. **(D)** Final view after completion of DRAF III procedure from the right nasal cavity with a 30° optic.

**FIGURE 15:**
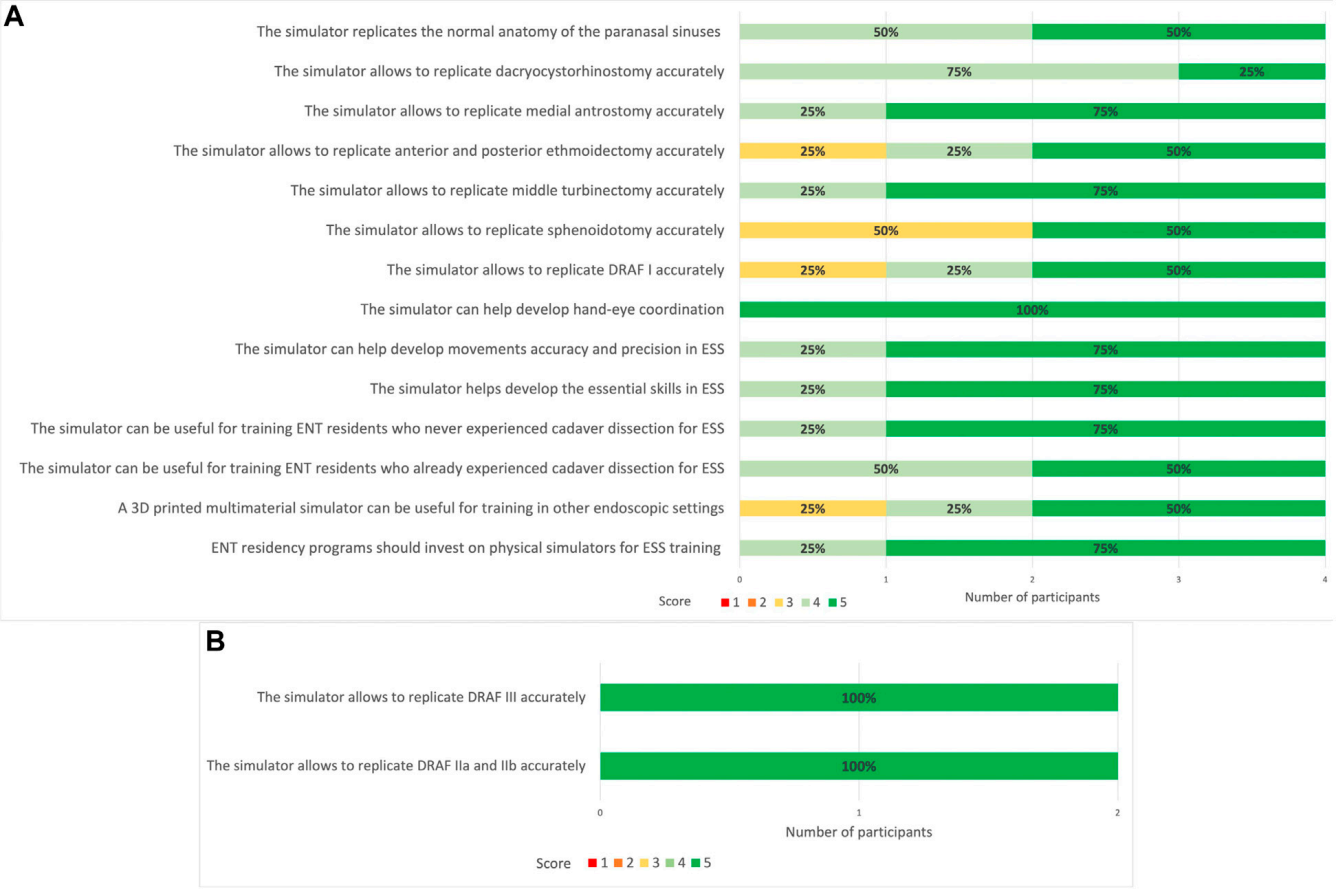
**(A)** 5-point Likert scale ratings for each question given by all participants (ENT experts). 1: strongly disagree; 2: disagree; 3: neutral; 4: agree; 5: strongly agree. **(B)** 5-point Likert scale ratings for DRAF II a, b and DRAF III given by the two experts. 1: strongly disagree; 2: disagree; 3: neutral; 4: agree; 5: strongly agree. In bold are reported the percentages for each rating given by the different members of the groups.

All 4 ENT experts were able to realize the required steps on the simulator with standard instruments set for ESS and drill (Figures 13, 14). Then they answered the 15-question survey, whose percentage results are summarized in Figure 15A.

Similar to the results obtained for the face and anatomical content validity questionnaire, all participants in this phase agreed or strongly agreed regarding the anatomical fidelity of the paranasal sinuses. Dacryocystorhinostomy, medial antrostomy and turbinectomy were rated as accurately replicable on the simulator. Also for ethmoidectomy and DRAF I the 75% of participants agreed or strongly agreed for accurate replication. The replication of sphenoidotomy, on the contrary, received more neutral ratings.

Moreover, both the experts performing DRAF IIa, b and III, strongly agreed that the model could accurately replicate these procedures (Figure 15B).

## 4 Discussion

The increasing number of medical trainees compared to the limited availability of body donors, and the ethical and biological issues related to ex-vivo dissection, have promoted research on alternative training methods to traditional cadaver dissection. Simulation-based training with 3D printed models has been favorably accepted in different surgical specialties as they provide an additional hands-on experience to improve anatomy learning, simulate surgical procedures in a risk-free setting, and eventually improve technical and cognitive skills (Meyer-Szary et al., 2022).

Regarding endoscopic sinus surgery (ESS), the training has been traditionally performed either on ovine models or cadaveric heads, which both lack from pathology and specific anatomical variants, and could be dissected just once. The development of synthetic models for ESS training has encountered several difficulties in relation to the replication of the complex anatomy and of the variable haptic feedback from different parts of the nose and paranasal sinuses.

The first low-fidelity models date back to 10 years ago, and despite having been demonstrated to improve basic endoscopic skills, the lack of anatomical and haptic fidelity hampered the possibility to simulate ESS procedures (Wais et al., 2012; Chang et al., 2017). More recently, Hsieh et al. (2018) developed a mono-material 3D printed endoscopic sinus and skull base surgical model with reported high anatomical and haptic accuracy, despite the lack of soft tissues (Hsieh et al., 2018). Zhuo et al. (2019) were the first to provide a pediatric nasal cavity and a post-surgical paranasal sinuses model, with positive results on anatomic realism and tactile feedback. However, the models were created by assembling several cuboid components subsequently fixed with double-sided tape so that only non-destructive tasks were simulated on their model, preventing its validation for ESS procedures. On the contrary, Alrasheed et al. (2017) developed and validated a 3D printed multi-material model for basic endoscopic skill training, as well as uncinectomy, anterior ethmoidectomy and frontal sinus dissection. The models had high anatomical fidelity and proved realistic haptic feedback, especially for bony dissection. Although soft structures were reproduced, the weakest feature reported was the replication of the mucosa. Alwani et al. (2020) validated the PHACON Sinus Trainer, one of the ESS simulators available on the market. Unlike the previous models, this simulator received low scores for the anatomical fidelity of both the mucosa and the bone, with only 30% and 42% of the participants rating them realistic, respectively.

The unmet needs of 3D printed simulators for ESS encouraged our group to develop a 3D printed multi-material and multi-color patient-specific simulator for ESS, using the Polyjet printing technology. The potentiality and the precision of this recent technology allowed us to create a model with bony and soft structures simultaneously, reaching a high level of anatomical fidelity and an overall satisfactory realistic haptic feedback, as suggested by our results. Indeed, the technology has many benefits, including excellent resolution (up to 0.016 mm), smooth surfaces, and the ability to print multi-material and multi-color parts, thanks to its multiple print heads. Furthermore, it is possible to dynamically mix materials and create “digital materials” with new characteristics. Indeed, it is possible to print rubber-like materials targeting a specific Shore-A hardness, as well as to mix up rigid and rubber-like materials, creating new materials with hybrid properties.

However, we decided not to use the rubber-like Agilus Stratasys material, which has a minimum achievable Shore-A 30, to reproduce the facial skin, since preliminary tests with surgeons confirmed that the obtained 3D printed part is still too rigid if compared to the real facial skin. Moreover, using Agilus material at the minimum Shore-A 30, the parts can be printed only in white, so it is not possible to achieve a realistic color for the skin. Many papers in the literature report the use of the Dragon Skin FX-Pro, especially for the replication of the human skin using specific pigments to have different realistic skin coloration (Francesconi et al., 2015; Teyssier et al., 2019; Ock et al., 2020; Ock et al., 2021).

Regarding the turbinates, we decided to fill them with silicone because the filling with Agilus Shore-A 30 made them too rigid. Conversely, the silicone filling allows the turbinates to be more compressible even if they still maintain a greater elasticity if compared to the real ones.

Moreover, the silicone has realistic feedback to cut, and we decided to use it also for simulating the nasal mucosa; also in this case, using an additional layer of Agilus Shore-A 30 on the nasal septum is not a good solution to achieve a realistic replication of the mucosa.

In our simulator, we were able to replicate the mucosa on the nasal septum so that the simulation of mucosal incision and mucoperichondrial flap elevation could be successfully performed on the 3D printed model by all participants. Alreefi et al., 2017 were the first to propose a training model for endoscopic septoplasty, using the TangoPlus FLX930 material from Stratasys to replicate the nasal mucosa. The procedure was scored as realistic, but the haptic feedback and membrane coloring received the lowest ratings. In our model, silicone was used to manually cover the nasal cartilage, obtaining a soft layer that could be realistically incised with a sickle knife or scalpel and a tissue-to-tissue surface dissectible with a Freer elevator.

To the best of our knowledge, this study is the first to consider the feasibility of endoscopic lacrimal surgery on a 3D printed model. The nasolacrimal duct and sac were realized with a different material (VeroYellow and Agilus30 with Shore-A 35), obtaining a color quite similar to the surrounding bone, as it is in real anatomy. The consistency of the sac resulted quite fragile so that in one case it was accidentally drilled before identifying it.

Moreover, in our model some high-risk anatomical landmarks such as the lamina papyracea and the dura mater of the anterior skull base were replicated with layers of material that, despite differences in color from the real structures, resulted not easily identifiable during endoscopic dissection, as in a real-life situation during ESS (both *in vivo* and on cadaver).

We also provide the replication of the interface between the endonasal structures and the orbital and intracranial content. Such boundaries are fundamental in understanding the limits of anatomical dissection and in preventing major complications of ESS, thus their reproduction should be as accurate as possible. Regarding this, the anatomical fidelity of our model was confirmed by the results of the surveys.

Our efforts to fabricate an increasingly realistic patient-specific simulator are in line with another recent experience of advanced ENT simulators based on 3D printing technology (Suzuki et al., 2021; Suzuki et al., 2022) which demonstrates how this kind of training tool may be beneficial to practice on difficult anatomies such as the ones in ESS field.

The introduction of AR feature may have an incremental value for the simulator, since it combines the benefits of virtual and physical tools.

Other than its educational role in facilitating the understanding of the anatomy of the sino-nasal district, this application could be of particular interest in the surgical planning of complex ESS procedures, where the navigation across a pathological model could help the surgeon to decide upon a specific endoscopic approach and possibly to foresee critical intraoperative steps to avoid complications.

According to the face and anatomical content validity assessment, our simulator showed anatomic realism and utility in learning anatomy and developing psychomotor skills. We noticed that across the simulations of non-destructive tasks, the silicone of the facial skin changed its consistency resulting softer and softer. The neutral positions of some users in judging the haptic feedback of the nostrils and nose tip may be attributed to the first attempts on the model. Similarly, the realism in using the instruments and endoscope inside the nasal cavity received heterogeneous scores. Other than the changing rigidity of the external nose, an influencing factor could be the impossibility to fracture the turbinates and/or the nasal septum, and to use decongestant agents to enlarge the endonasal space, as routinely performed during ESS. The turbinates showed greater elasticity than the real structures; this caused a more difficult passage of the endoscope and instruments, either lateral or medial to them, inside the nasal cavity due to the spring back effect of the elastic material.

Overall, the operative time for non-destructive tasks was longer for residents than the experts as previously reported by other studies (Alrasheed et al., 2017; Zhuo et al., 2019). However, this difference was not significant, probably for the paucity of participants.

Ethmoidectomy and sphenoidotomy were scored more variably than other ESS steps among the experts, according to the task-specific validation questionnaire. Reviewing these results and the video recording of the procedures, we found that the inferior scores were given from the two experts dissecting the right sides. A higher number of ethmoidal cells filled with the printing support material were found on the right ethmoid compared to the contralateral. This made the dissection of this area more difficult and longer than in real ESS. Since both right-side dissectors chose to perform transethmoidal sphenoidotomy, these participants rated the access to the sphenoid less realistic. On the opposite side, sphenoidotomy which was performed through a parasettal route by one participant and transethmoidal route by the other, received the highest score in terms of replication.

The experts agreed on the utility of the simulator during ENT residency, both for those naïve and those with some experience in cadaveric dissection, and confirmed the idea of including this kind of training tools in ENT residency programs.

The simulator we developed is primarily addressed to provide a training tool for ESS procedures mainly for residents, as an alternative or complementary solution to cadaveric dissection.

For this primary intended use, our idea is to select various exemplary cases of normal and pathologic sino-nasal anatomies and to reconstruct from them exemplary simulators that can be used by residents for ESS training at different levels of complexity. Therefore, the simulators are fabricated starting from patient-specific anatomy, thus requiring time for patient data collection, 3D model reconstruction, and then printing. However, in this case, the short production time is not a strict requirement as in the case of using the simulator for preoperative rehearsal the day before going to the operating room.

In the future, it could be interesting to foresee producing, for complex surgical cases, a patient-specific simulator to be used by the surgeon for preoperative rehearsal before surgery. In that case, surely the whole process should be speeded up.

The average time it took to obtain the current ESS simulator was about 16 h for the patient-specific anatomical part (8 h for image segmentation/3D model refinement and 8 h for printing process and cleaning), and about 20 h for the design and printing of the external box and face mold.

A strategy to reduce these times is to take advantage of the modular design of the simulator, in which the internal part relating to the patient-specific paranasal sinuses anatomy can be changed and customized from time to time, while the external part (box and face mold) can be reused.

### 4.1 Limitations

Despite all our efforts in the cleaning of the paranasal sinuses model, some residual printing support material remained inside some ethmoidal cells, reducing the anatomical reliability and the performance of selected ESS steps. Future studies will include improvements in the design of the model to entirely clean it and reproduce the variable degree of pneumatization of the paranasal sinuses.

The performance of ESS procedures was evaluated by a subjective Likert scale applied to not-validated questionnaires. However, for the purposes of this preliminary study, these methods could be considered acceptable in identifying positive feedback from the users. This limitation is shared with several other validation studies on the same topic (Alrasheed et al., 2017; AlReefi et al., 2017).

Our results are promising but the evaluation was performed on a restricted number of participants.

A future study will be planned to compare the ESS training based on our simulator and the one based on cadaver dissection, involving a higher number of trainees.

The AR feature we introduced can certainly be improved, e.g. by implementing the possibility to manipulate the virtual objects in addition to making them visible or not. Although we have received enthusiastic feedback from surgeons who have also experimented with the AR feature, its educational and training benefits must be more systematically evaluated.

## 5 Conclusion

This study demonstrated that a detailed multi-material 3D printed model of sinus anatomy can be generated with a high level of anatomical accuracy and an overall satisfactory haptic response. This technology has the potential to be used in surgical training of ESS procedures, as an alternative or complementary training solution to cadaveric dissection. It could also potentially play a significant role in preoperative planning, since it may provide an individualized preoperative design on which the surgeon can rehearse before surgery, to shorten operation duration and ensure safe and effective surgery in patients.

## Data Availability

The raw data supporting the conclusions of this article will be made available by the authors, without undue reservation.

## References

[B1] Acar B. Gunbey E. Babademez M. A. Karabulut H. Gunbey H. P. Karasen R. M. (2010). Utilization and dissection for endoscopic sinus surgery training in the residency program. J. Craniofac. Surg. 21, 1715–1718. 10.1097/SCS.0b013e3181f3c73b 21119406

[B2] Alrasheed A. S. Nguyen L. H. P. Mongeau L. Funnell W. R. J. Tewfik M. A. (2017). Development and validation of a 3D-printed model of the ostiomeatal complex and frontal sinus for endoscopic sinus surgery training: 3D-printed endoscopic sinus surgery simulator. Int. Forum Allergy Rhinol. 7, 837–841. 10.1002/alr.21960 28614638

[B3] AlReefi M. A. Nguyen L. H. P. Mongeau L. G. Haq B. Boyanapalli S. Hafeez N. (2017). Development and validation of a septoplasty training model using 3-dimensional printing technology: Septology training model with 3D printing. Int. Forum Allergy Rhinol. 7, 399–404. 10.1002/alr.21887 27897397

[B4] Alwani M. M. Svenstrup T. J. Bandali E. H. Sharma D. Higgins T. S. Wu A. W. (2020). Validity testing of a three-dimensionally printed endoscopic sinonasal surgery simulator. Laryngoscope 130, 2748–2753. 10.1002/lary.28356 31714604

[B5] Battaglia S. Badiali G. Cercenelli L. Bortolani B. Marcelli E. Cipriani R. (2019). Combination of CAD/CAM and augmented reality in free fibula bone harvest. Plastic Reconstr. Surg. - Glob. Open 7, e2510. 10.1097/GOX.0000000000002510 PMC6908345 31942302

[B6] Beale T. J. Madani G. Morley S. J. (2009). Imaging of the paranasal sinuses and nasal cavity: Normal anatomy and clinically relevant anatomical variants. Seminars Ultrasound, CT MRI 30, 2–16. 10.1053/j.sult.2008.10.011 19388234

[B7] Bianchi L. Barbaresi U. Cercenelli L. Bortolani B. Gaudiano C. Chessa F. (2020). The impact of 3D digital reconstruction on the surgical planning of partial nephrectomy: A case-control study. Still time for a novel surgical trend? Clin. Genitourin. Cancer 18, e669–e678. 10.1016/j.clgc.2020.03.016 32354617

[B8] Bianchi L. Schiavina R. Barbaresi U. Angiolini A. Pultrone C. V. Manferrari F. (2019). 3D Reconstruction and physical renal model to improve percutaneous punture during PNL. Int. braz J. Urol. 45 (6), 1281–1282. 10.1590/S1677-5538.IBJU.2018.0799 31408285 PMC6909851

[B9] Cabibihan J.-J. (2011). Patient-specific prosthetic fingers by remote collaboration–A case study. PLoS ONE 6, e19508. 10.1371/journal.pone.0019508 21573246 PMC3087799

[B10] Cercenelli L. De Stefano A. Billi A. M. Ruggeri A. Marcelli E. Marchetti C. (2022). AEducaAR, anatomical education in augmented reality: A pilot experience of an innovative educational tool combining AR technology and 3D printing. Int. J. Environ. Res. Public Health 19 (3), 1024. 10.3390/ijerph19031024 35162049 PMC8834017

[B11] Chang D. R. Lin R. P. Bowe S. Bunegin L. Weitzel E. K. McMains K. C. (2017). Fabrication and validation of a low-cost, medium-fidelity silicone injection molded endoscopic sinus surgery simulation model: Silicone Injection Molded ESS Model. Laryngoscope 127, 781–786. 10.1002/lary.26370 28000224

[B12] Francesconi M. Freschi C. Sinceri S. Carbone M. Cappelli C. Morelli L. (2015). New training methods based on mixed reality for interventional ultrasound: Design and validation. Annu. Int. Conf. IEEE Eng. Med. Biol. Soc. 2015, 5098–5101. 10.1109/EMBC.2015.7319538 26737438

[B13] Ganguli A. Pagan-Diaz G. J. Grant L. Cvetkovic C. Bramlet M. Vozenilek J. (2018). 3D printing for preoperative planning and surgical training: A review. Biomed. Microdevices 20, 65. 10.1007/s10544-018-0301-9 30078059

[B14] Griffin M. F. Premakumar Y. Seifalian A. M. Szarko M. Butler P. E. M. (2016). Biomechanical characterisation of the human nasal cartilages; implications for tissue engineering. J. Mat. Sci. Mat. Med. 27, 11. 10.1007/s10856-015-5619-8 PMC4681753 26676857

[B15] Hsieh T. Cervenka B. Dedhia R. Strong E. B. Steele T. (2018). Assessment of a patient-specific, 3-dimensionally printed endoscopic sinus and skull base surgical model. JAMA Otolaryngol. Head. Neck Surg. 144, 574. 10.1001/jamaoto.2018.0473 29799965 PMC6145784

[B16] Humphreys I. M. Hwang P. H. (2015). Avoiding complications in endoscopic sinus surgery. Otolaryngologic Clin. N. Am. 48, 871–881. 10.1016/j.otc.2015.05.013 26117296

[B17] Jahya A. Herink M. Misra S. (2013). A framework for predicting three-dimensional prostate deformation in real time: Predicting 3D prostate deformation in real time. Int. J. Med. Robot. Comput. Assist. Surg. 9, e52–e60. 10.1002/rcs.1493 23495193

[B18] Keerl R. Stankiewicz J. Weber R. Hosemann W. Draf W. (1999). Surgical experience and complications during endonasal sinus surgery. Laryngoscope 109, 546–550. 10.1097/00005537-199904000-00005 10201738

[B19] Laeeq K. Lin S. Y. Varela D. A. D. V. Lane A. P. Reh D. Bhatti N. I. (2013). Achievement of competency in endoscopic sinus surgery of otolaryngology residents: Competency in Endoscopic Sinus Surgery. Laryngoscope 123, 2932–2934. 10.1002/lary.23509 24122507

[B20] Mayer R. Liacouras P. Thomas A. Kang M. Lin L. Simone C. B. (2015). 3D printer generated thorax phantom with mobile tumor for radiation dosimetry. Rev. Sci. Instrum. 86, 074301. 10.1063/1.4923294 26233396

[B21] Meyer-Szary J. Luis M. S. Mikulski S. Patel A. Schulz F. Tretiakow D. (2022). The role of 3D printing in planning complex medical procedures and training of medical professionals—cross-sectional multispecialty review. Int. J. Environ. Res. Public Health 19, 3331. 10.3390/ijerph19063331 35329016 PMC8953417

[B22] Ock J. Gwon E. Kim D. Kim S. Kim N. (2020). Patient-specific and hyper-realistic phantom for an intubation simulator with a replaceable difficult airway of a toddler using 3D printing. Sci. Rep. 10, 10631. 10.1038/s41598-020-67575-5 32606342 PMC7326915

[B23] Ock J. Kim T. Lee S. Yang T. S. kim M. Jeong W. (2021). Evaluation of skin cancer resection guide using hyper-realistic *in-vitro* phantom fabricated by 3D printing. Sci. Rep. 11, 8935. 10.1038/s41598-021-88287-4 33903639 PMC8076220

[B24] Richmon J. D. Sage A. B. Wong V. W. Chen A. C. Pan C. Sah R. L. (2005). Tensile biomechanical properties of human nasal septal cartilage. Am. J. Rhinol. 19, 617–622. 10.1177/194589240501900616 16402652

[B25] Richmon J. D. Sage A. Wong V. W. Chen A. C. Sah R. L. Watson D. (2006). Compressive biomechanical properties of human nasal septal cartilage. Am. J. Rhinology 20, 496–501. 10.2500/ajr.2006.20.2932 17063745

[B26] Sander I. Liepert T. Doney E. Leevy W. Liepert D. (2017). Patient education for endoscopic sinus surgery: Preliminary experience using 3D-printed clinical imaging data. J. Funct. Biomater. 8, 13. 10.3390/jfb8020013 28387702 PMC5491994

[B27] Schiavina R. Bianchi L. Borghesi M. Chessa F. Cercenelli L. Marcelli E. (2019). Three-dimensional digital reconstruction of renal model to guide preoperative planning of robot-assisted partial nephrectomy. Int. J. Urol. 26, 931–932. 10.1111/iju.14038 31234241

[B28] Stammberger H. Kennedy D. W. Bolger W. (1995). Paranasal sinuses: Anatomic terminology and nomenclature. Ann. Otol. Rhinol. Laryngol. 167, 17–16. 10.1177/000348949510410s01 7574267

[B29] Stew B. Kao S. S.-T. Dharmawardana N. Ooi E. H. (2018). A systematic review of validated sinus surgery simulators. Clin. Otolaryngol. 43, 812–822. 10.1111/coa.13052 29247602

[B30] Suzuki M. Vyskocil E. Ogi K. Matoba K. Nakamaru Y. Homma A. (2021). Remote training of functional endoscopic sinus surgery with advanced manufactured 3D sinus models and a telemedicine system. Front. Surg. 8, 746837. 10.3389/fsurg.2021.746837 34660685 PMC8517106

[B31] Suzuki M. Me K. M. Watanabe R. Suzuki T. Matoba K. Nakazono A. (2022). Repetitive simulation training with novel 3D-printed sinus models for functional endoscopic sinus surgeries. Laryngoscope. Investig. Otolaryngol. 7, 943–954. 10.1002/lio2.873 PMC9392405 36000044

[B32] Tack P. Victor J. Gemmel P. Annemans L. (2016). 3D-printing techniques in a medical setting: A systematic literature review. Biomed. Eng. OnLine 15, 115. 10.1186/s12938-016-0236-4 27769304 PMC5073919

[B33] Tejo-Otero A. Buj-Corral I. Fenollosa-Artés F. (2020). 3D printing in medicine for preoperative surgical planning: A review. Ann. Biomed. Eng. 48, 536–555. 10.1007/s10439-019-02411-0 31741226

[B34] Teyssier M. Bailly G. Pelachaud C. Lecolinet E. Conn A. Roudaut A. (2019). “Skin-on interfaces: A bio-driven approach for artificial skin design to cover interactive devices,” in Proceedings of the 32nd Annual ACM Symposium on User Interface Software and Technology, 17 October 2019 (New Orleans LA USA: ACM), 307–322. 10.1145/3332165.3347943

[B35] Vaid S. Vaid N. (2015). Normal anatomy and anatomic variants of the paranasal sinuses on computed tomography. Neuroimaging Clin. N. Am. 25, 527–548. 10.1016/j.nic.2015.07.002 26476378

[B36] Wais M. Ooi E. Leung R. M. Vescan A. D. Lee J. Witterick I. J. (2012). The effect of low-fidelity endoscopic sinus surgery simulators on surgical skill. Int. Forum Allergy & Rhinology 2, 20–26. 10.1002/alr.20093 22311837

[B37] Weber R. K. Hosemann W. (2015). Comprehensive review on endonasal endoscopic sinus surgery. GMS Curr. Top. Otorhinolaryngol. Head. Neck Surg. 14, Doc08. 10.3205/cto000123 26770282 PMC4702057

[B38] Zhuo C. Lei L. Yulin Z. Wentao L. Shuangxia W. Chao W. (2019). Creation and validation of three-dimensional printed models for basic nasal endoscopic training. Int. Forum Allergy Rhinol. 9, 695–701. 10.1002/alr.22306 30748103

