# Supplementary material for: Assessment of a novel patient-specific 3D printed multi-material simulator for endoscopic sinus surgery
Source: Front Bioeng Biotechnol. 2022 Nov 17;10:974021. doi: 10.3389/fbioe.2022.974021 (PMC9712453; doi:10.3389/fbioe.2022.974021)
Supplement: Supplementary file 3 [file Table1.DOCX]

**Survey**

**Answer each question with an “X” in the corresponding cell:**

1. Strongly disagree
2. Disagree
3. Neutral
4. Agree
5. Strongly agree

|  | **1** | **2** | **3** | **4** | **5** |
| --- | --- | --- | --- | --- | --- |
| 1. The simulator replicates the normal anatomy of nasal cavities |  |  |  |  |  |
| 1. Haptic feedback of the bony structures is realistic |  |  |  |  |  |
| 1. Haptic feedback of the turbinates is realistic |  |  |  |  |  |
| 1. Haptic feedback of the nasal septum is realistic |  |  |  |  |  |
| 1. Haptic feedback of the nostrils and the nose tip is realistic |  |  |  |  |  |
| 1. The use of instruments and the endoscope inside the nasal cavity is realistic |  |  |  |  |  |
| 1. The simulator can be useful for learning anatomy |  |  |  |  |  |
| 1. The simulator can help develop hand-eye coordination |  |  |  |  |  |
| 1. The simulator can help develop movements accuracy and precision in endonasal endoscopy |  |  |  |  |  |
| 1. A 3D printed multi-material simulator can be useful for training in other endoscopic settings |  |  |  |  |  |
