# Supplementary material for: Assessment of a novel patient-specific 3D printed multi-material simulator for endoscopic sinus surgery
Source: Front Bioeng Biotechnol. 2022 Nov 17;10:974021. doi: 10.3389/fbioe.2022.974021 (PMC9712453; doi:10.3389/fbioe.2022.974021)
Supplement: Supplementary file 4 [file Table2.DOCX]

**Survey**

**For each question, answer with an “X” in the corresponding cell:**

1. Strongly disagree
2. Disagree
3. Neutral
4. Agree
5. Strongly agree

|  | **1** | **2** | **3** | **4** | **5** |
| --- | --- | --- | --- | --- | --- |
| 1. The simulator replicates the normal anatomy of the paranasal sinuses |  |  |  |  |  |
| 1. The simulator allows to replicate dacryocystorhinostomy accurately |  |  |  |  |  |
| 1. The simulator allows to replicate medial antrostomy accurately |  |  |  |  |  |
| 1. The simulator allows to replicate anterior and posterior ethmoidectomy accurately |  |  |  |  |  |
| 1. The simulator allows to replicate middle turbinectomy accurately |  |  |  |  |  |
| 1. The simulator allows to replicate sphenoidotomy accurately |  |  |  |  |  |
| 1. The simulator allows to replicate DRAF I accurately |  |  |  |  |  |
| 1. The simulator allows to replicate DRAF IIa and IIb accurately |  |  |  |  |  |
| 1. The simulator allows to replicate DRAF III accurately |  |  |  |  |  |
| 1. The simulator can help develop hand-eye coordination |  |  |  |  |  |
| 1. The simulator can help develop movements accuracy and precision in ESS |  |  |  |  |  |
| 1. The simulator helps develop the essential skills in ESS |  |  |  |  |  |
| 1. The simulator can be useful for training ENT residents who never experienced cadaver dissection for ESS |  |  |  |  |  |
| 1. The simulator can be useful for training ENT residents who already experienced cadaver dissection for ESS |  |  |  |  |  |
| 1. A 3D printed multi-material simulator can be useful for training in other endoscopic settings |  |  |  |  |  |
| 1. ENT residency programs should invest on physical simulators for ESS training |  |  |  |  |  |
